# Clustered warming tolerances and the nonlinear risks of biodiversity loss on a warming planet

**DOI:** 10.1098/rstb.2023.0321

**Published:** 2025-01-09

**Authors:** Joseph Williamson, Muyang Lu, M. Florencia Camus, Richard D. Gregory, Ilya M. D. Maclean, Juan C. Rocha, Marjo Saastamoinen, Robert J. Wilson, Jon Bridle, Alex L. Pigot

**Affiliations:** ^1^ Department of Genetics, Evolution and Environment, University College London, London WC1E 6BT, UK; ^2^ College of Life Science, Sichuan University, Chengdu 610065, China; ^3^ RSPB Centre for Conservation Science, Sandy, Bedfordshire SG19 2DL, UK; ^4^ Environment & Sustainability Institute, University of Exeter, Penryn Campus, Exeter TR10 9FE, UK; ^5^ The Anthropocene Laboratory, Royal Swedish Academy of Sciences, Stockholm 114 18, Sweden; ^6^ Stockholm Resilience Centre, Stockholm University, Stockholm 106 91, Sweden; ^7^ Research Centre for Ecological Change, Organismal and Evolutionary Biology Research Programme, Faculty of Biological and Environmental Sciences, University of Helsinki, Helsinki 00014, Finland; ^8^ Department of Biogeography and Global Change, Museo Nacional de Ciencias Naturales, Madrid 28006, Spain

**Keywords:** climate change, global change, biodiversity loss, thermal limit, tipping point, thermal safety margin

## Abstract

Anthropogenic climate change is projected to become a major driver of biodiversity loss, destabilizing the ecosystems on which human society depends. As the planet rapidly warms, the disruption of ecological interactions among populations, species and their environment, will likely drive positive feedback loops, accelerating the pace and magnitude of biodiversity losses. We propose that, even without invoking such amplifying feedback, biodiversity loss should increase nonlinearly with warming because of the non-uniform distribution of biodiversity. Whether these non-uniformities are the uneven distribution of populations across a species’ thermal niche, or the uneven distribution of thermal niche limits among species within an ecological community, we show that in both cases, the resulting clustering in population warming tolerances drives nonlinear increases in the risk to biodiversity. We discuss how fundamental constraints on species’ physiologies and geographical distributions give rise to clustered warming tolerances, and how population responses to changing climates could variously temper, delay or intensify nonlinear dynamics. We argue that nonlinear increases in risks to biodiversity should be the null expectation under warming, and highlight the empirical research needed to understand the causes, commonness and consequences of clustered warming tolerances to better predict where, when and why nonlinear biodiversity losses will occur.

This article is part of the discussion meeting issue ‘Bending the curve towards nature recovery: building on Georgina Mace’s legacy for a biodiverse future’.

## 1. Introduction

Global biodiversity is in steep decline, driven largely by land-use change, human exploitation and invasive species [1–4]. These losses are impacting human societies, compromising prosperity, health and sustainability [5]. A major challenge for the coming years is to halt and reverse this decline, to ‘bend the curve on biodiversity loss’ to an upward trajectory [6,7]. Targets to protect and restore biodiversity were agreed by the Conference of the Parties to the Convention on Biological Diversity in 2023 (https://www.cbd.int/gbf/). However, these ambitious goals could be undermined by the growing impacts of rapid, human-driven climate change [8].

At approximately 1.3°C of global warming since the pre-industrial period, the effects of climate change on biodiversity are already pervasive, adversely impacting ecosystems and regions across the planet [8,9]. Biodiversity projections indicate that our current warming trajectory is likely to have catastrophic impacts on species and the ecosystems they compose, with 20–30% of species projected to be at critical risk of extinction at a global warming level of 2.5°C [8,10]. However, these projections involve major uncertainties, prominent among these being our limited understanding of how ecological communities will respond to changing climates [11]. A key outstanding question is whether the risks to species, ecological communities and their genetic and functional diversity are likely to increase linearly with the magnitude of warming. Or should we expect biodiversity losses to increase nonlinearly, accelerating upwards beyond critical levels of warming [10]? If biodiversity responses are nonlinear, where and at what level of warming will they occur for different populations, species and ecological communities [10,12]? Such questions echo the broader debate in ecology concerning how often ecological systems exhibit threshold responses to environmental change and whether these can be reliably predicted in advance [13,14]. Answering these questions is critical in efforts to conserve biodiversity and manage ecosystems, to define dangerous levels of climate warming for ecological resilience [15] and, more broadly, to estimate the planetary boundaries within which humanity and complex ecosystems can continue to thrive [5,16–18].

Most biological rates and performance metrics of individual species respond nonlinearly to changes in temperature (e.g. [19]). For example, ectotherms generally exhibit a skewed unimodal thermal performance curve that rises gradually to an optimum before steeply declining towards the upper critical thermal limit [20]. These nonlinear responses at the level of individuals of given species have been shown to drive nonlinear trends in species’ abundance [21,22] and in population extinction risk [23]. Moreover, dynamic ecological feedbacks that buffer systems against external forcing can break down as stressors increase, leading to nonlinear ‘tipping points’ in biodiversity loss [24–29]. For example, climate-driven population declines can cause the loss of neighbouring subpopulations that are maintained by immigration, or the coextinctions of other species linked by ecological dependencies [16,30]. At the level of ecosystems and biomes, feedback loops between organisms and their physical environment can drive abrupt and irreversible transitions between different ecological states (i.e. regime shifts). For example, declining transpiration rates driven by forest loss and climate change across the Amazon Basin are projected to cause further drought-induced dieback, eventually leading to regional collapse of the forest system [31,32].

It is widely appreciated that dynamic feedbacks between the different biological units that compose biodiversity—whether they be individuals, populations or species—can drive nonlinear ecological responses and increased risks to biodiversity under climate change. But are these dynamic feedbacks required for biodiversity to respond nonlinearly to environmental change? How would the magnitude of biodiversity loss increase in a world in which such feedbacks were absent? Understanding such an expectation would provide insight into the causes of abrupt ecological responses in the past, help estimate the prospects of nonlinear changes in the future, and direct ways that nonlinear (and abrupt) changes in ecological communities could be avoided or reversed.

**Figure 1.**
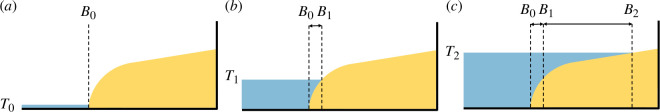
The nonlinear inundation of a beach as the tide gradually rises provides an analogy for how risks to biodiversity can accelerate under steady climate warming. At time point 0 (*a*) *B*_0_ denotes the initial extent of the beach while *T*_0_ denotes the initial sea level. At time point 1 (*b*) sea levels have risen by a set amount to *T*_1_, but only a small area of the beach is flooded (*B*_0_ *− B*_1_). At time point 2 (*c*) sea levels have risen by the same amount again, but larger areas of beach have been inundated (*B*_1_ *- B*_2_ » *B*_1_ *−B*_0_). While here we use the nonlinear increase in the area at risk from the rising tide as an analogy, the exposure of biodiversity [33] and human populations [34] to rising sea levels could represent a real example of accelerating risk under climate change without invoking any feedback loops. This is because most land area, and therefore terrestrial wildlife and human populations, are concentrated at low elevations.

**Figure 2.**
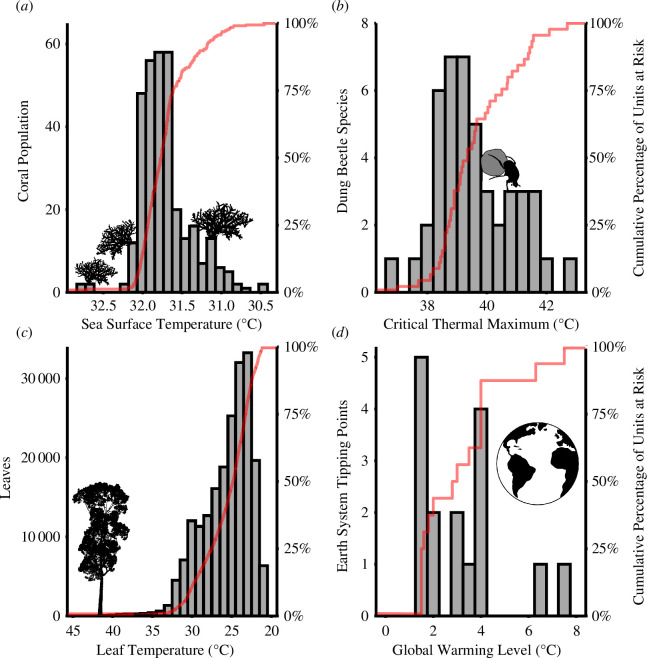
Examples of the risk of nonlinear biodiversity responses to temperature at different scales of biological organization. Frequency distribution of (*a*) current sea surface temperatures across the geographical distribution of the coral *Pectinia pygmaea* [35], (*b*) critical thermal limits (maxima) of dung beetle species within a tropical forest assemblage [36], (*c*) leaf temperatures through time within a tropical forest tree [37], and (*d*) global warming levels at which biological and physical tipping elements in major Earth systems are at risk of being triggered [38]. Red lines denote the corresponding cumulative distributions, showing the nonlinear increase in the percentage of biological units exposed to temperatures beyond their thermal limits as the climate warms. Red lines are nonlinear owing to (*a*,*c*) the clustering of biological units that share the same thermal limit across a temperature gradient or (*b*,*d*) the clustering of thermal limits of biological units (and physical units in (*d*)). Graphs (*a*,*b*) demonstrate the nonlinear risk posed by clustered warming tolerances to a species across its geographical range and across species within an ecological community, respectively, and are the focus of this manuscript. In all cases (*a*–*d*), nonlinear risks are driven by clustered warming tolerances (i.e. the magnitude of warming before a biological unit—be that a population, a species within an assemblage, an organ within an individual, or a major Earth system—is committed to collapse). For ease of visualization, in (*a*,*c*) the temperature axis is reversed (i.e. warmer temperatures on the left) so that in all panels cumulative risk increases from left to right with warming. Tree silhouette image credit is given to T. Michael Keesey from a photo by Bernard Dupont (licence https://creativecommons.org/licenses/by-sa/3.0/).

To visualize nonlinear responses without ecological feedbacks, imagine standing on a beach as the tide rises (figure 1). At first, owing to the steep front of the beach, only small areas are inundated by the rising sea. However, as the tide rises further, the progressively shallower gradient of the beach allows the sea to rush in, suddenly submerging large areas. Even though the tide rises at a constant rate, a nonlinear vertical profile of the beach, regardless of whether this is convex, concave or more complex in shape, results in a nonlinear increase in the area inundated. In the same way, a linear warming trend (or change in another abiotic driver) could drive a nonlinear increase in its effects on biodiversity simply because the biological units that constitute biodiversity are unevenly distributed. For a single species, this can be an uneven distribution of populations across the thermal gradients within its range (figure 2*a*), while for an assemblage, this can be an uneven distribution of species’ thermal limits in niche space (figure 2*b*).

Here, we explore how the non-uniform distribution of populations of a species across a thermal gradient, and species’ thermal limits across niche space, could drive nonlinear increases in risks to biodiversity with linear warming. Importantly, we show that this should occur even in a hypothetical world without ecological feedbacks, where populations or species do not interact with one another, or with the climate. We organize our discussion in the following sections. First, we review recent empirical and modelling studies [12,35] suggesting that risks to species’ geographical ranges and ecological assemblages will increase nonlinearly under climate change. We show that, in both these cases, this nonlinear increase in the risk of biodiversity losses can arise from the clustered warming tolerances of populations (i.e. the magnitude of warming before a population is committed to extinction), be they populations of a species across its geographical range, or populations of different species within a local ecological assemblage. Second, we discuss the potential causes of clustered warming tolerances across populations, suggesting that this could reflect fundamental biophysical constraints, operating from the molecular to the planetary level, on the geographical distribution and thermal tolerance of species. Third, given a null expectation that risks to species and ecological assemblages increase nonlinearly under climate warming, we discuss how adding ecological and evolutionary responses back into this model could alter the expected dynamics of biodiversity loss, either tempering or intensifying the nonlinear response expected by clustered warming tolerances. Although our discussion of clustered warming tolerances focuses on nonlinear increases in risk (i.e. number of populations and species exposed to unsuitable temperatures) as a function of the magnitude of long-term (e.g. decades to centuries) climate warming, we also discuss how this risk relates to responses to short-term climate pulses (e.g. heatwaves). Furthermore, while we focus on nonlinear increases in risk to species’ geographical ranges and ecological assemblages due to warming, we also touch on examples of nonlinearity driven by other abiotic variables and across different levels of biological organization, also without needing to invoke feedback mechanisms. Finally, we consider how advances in our understanding of the drivers, location and prevalence of clustered warming tolerances are necessary to inform the protection and recovery of biodiversity in an era of rapid anthropogenic climate change.

## 2. Nonlinear risks to species and ecological assemblages under global warming

Most projections of biodiversity under future climate change are based on estimating the geographical area of species’ ranges that will be exposed to conditions beyond the limits of their climatic niche, and so where local extinctions are expected to follow [39]. Many studies predict contractions of species’ ranges as suitable conditions disappear or shift across space at rates far exceeding the dispersal capacities of most species [10]. Pigot *et al.* [35] used annual resolution climate change projections to show that for thousands of terrestrial and marine species the area of their existing geographical distribution exposed to temperatures above their realized thermal limit (i.e. the maximum temperature historically experienced across a species’ range) will increase nonlinearly over the coming decades. In other words, while most populations (i.e. grid cells where a species occurs) initially remain below the species’ thermal limit, eventually a threshold of warming is passed beyond which large areas of a species’ geographical range are exposed to potentially dangerous temperatures, much as in our analogy the rising tide eventually submerges large areas of the beach following a small increase in sea level (figure 1). The abrupt increase in exposure described by Pigot *et al*. [35] could only partly be explained by abrupt increases in temperature. Instead, Pigot *et al*. [35] provided a novel mechanism, showing that while different populations across a species’ range experience different temperatures, this distribution is far from uniform, with most populations clustered over a relatively narrow range of temperatures, and therefore similarly vulnerable to warming (figure 2*a*). While this study focused on modelling the timing of thermal exposure, the results demonstrated that even linear increases in temperature could drive nonlinear (i.e. accelerating) increases in the area of species’ existing ranges at risk under climate change.

Using the same model but applied to ecological assemblages, Trisos *et al*. [12] showed that the number of co-occurring species exposed to temperatures beyond their upper thermal limits is also expected to increase abruptly over time under a constant rate of climate warming. Again, this could not be explained simply by rapid increases in temperature. Instead, the projected timing of exposure was synchronized because many co-occurring species share similar thermal limits. One caveat is that these projections were based on species’ realized thermal limits inferred from coarse geographical range maps, and thermal limits may cluster for artefactual reasons, including steep environmental gradients [40,41] or a truncation of species’ realized niches by geographical barriers [42], meaning that actual tolerances may be higher. However, these factors are unlikely to be sufficient, given that data gathered from experiments suggest that such clustering is also an empirical pattern found in the fundamental niche limits of co-occurring species.

One example of strong clustering of fundamental niche limits comes from Williamson *et al*. [36], who estimated the critical thermal limits of 45 dung beetle species that co-occur in a Bornean tropical forest. These limits ranged from 37 to 43°C, suggesting varying sensitivities to warming (figure 2*b*). Indeed, if species’ thermal limits were uniformly spaced between these bounds, then the number of species exposed to intolerable conditions would increase at a rate of approximately eight species (17%) per 1°C of warming. However, in reality, almost half (47%) of the species have upper critical limits clustered around 39°C (± 0.5°C). Similar patterns are observed in other locations and taxa. For example, despite a much broader range in thermal limits (>12°C) among the adults of co-occurring frog species at a site in Amazonia, 36% (18/50) of these thermal limits cluster at 38°C (± 1°C), double that expected if thermal limits were uniformly distributed between the observed bounds [43]. In two assemblages of subtropical tadpoles in South America, 56% (9/16) and 45% (5/11) of species had critical thermal limits within the range of a single degree celcius [44]. Similarly, *T*_50_ values (the temperature at which photosystem II functionality is reduced to 50%) for 42 co-occurring tree species at one site in Panama had a range of 7.5°C, but 62% (26/42) of these species were clustered at 50°C (± 1°C) [45]. Thus, a clustering of species thermal limits within assemblages appears widespread and reflects an empirical clustering of fundamental tolerances across co-occurring species, rather than just an artefact of using realized niches.

For both species’ ranges and ecological assemblages, we can consider the population of a species, here treated simply as the presence of a species at a particular location, as the fundamental biological unit at risk. Whether it be the clustered distribution of thermal limits among the different species composing an ecological assemblage, or the clustered distribution of populations across thermal gradients within a species’ geographical range, in both cases the abrupt increase in risk under warming arises from the clustered distribution of population ‘warming tolerances’—the differences between the conditions currently experienced by populations, and the temperature under which they would be committed to extinction [46]. Previous studies have demonstrated substantial global variation in the mean warming tolerance of species (and in the closely related concept of thermal safety margins) [47], often suggesting that tropical assemblages with narrower warming tolerances are at more immediate risk of warming [46,48]. However, rather than focusing on mean tolerances, here we emphasize how the non-uniform shape of the frequency distribution of population warming tolerances (across a species’ range or within an ecological assemblage) drives nonlinear increases in the risk of thermal exposure under warming (figure 2).

The idea that the clustering of warming tolerances leads to nonlinear forcing of biodiversity loss under sustained climate change has strong parallels to research linking the synchrony of population fluctuations under temporally variable climates to the stability of species’ regional abundances and ecological assemblages [49]. This ‘Moran effect’ means that, rather than varying independently, populations fluctuate synchronously across a species’ range owing to correlated changes in the environment [50], reducing the resilience of species’ occupancy. Many empirical studies have shown that synchrony in population fluctuations is a widespread phenomenon [51], with population fluctuations positively covarying over hundreds of kilometres [52], and that this phenomenon should increase the risk of species’ extinction [53]. Studies of the Moran effect have focused on demographic responses to weather variability (e.g. [54]). However, we suggest that a similar principle applies to explain the synchronized exposure of populations under sustained warming. Hansen *et al.* [50] highlight how baseline variation in thermal conditions across a species’ range will tend to decouple the dynamics of populations during heating events, because populations near the cold edge of the species’ range will increase in productivity while those at the hot edge will decrease. Although thermal conditions do vary across a species’ range, if, as we suggest, the general rule is for many populations to be clustered under similar thermal conditions (figure 2a) [35], then variation in the trajectories of population responses will also be tightly coupled, with many populations responding to warming in a coordinated way. This means that the clustering of population warming tolerances that cause synchrony in the commitment of populations to extinction under sustained warming provides a long-term analogue to the synchrony of population fluctuations under temporally variable weather.

At the level of ecological assemblages, although experimental studies have demonstrated the occurrence of compensatory dynamics [55], meta-analyses indicate that fluctuations in growth rate and abundance across co-occurring species are often positively correlated in nature [56,57]. This presumably arises because co-occurring species have been selected (or filtered) to have similar environmental niches. Ghosh *et al*. [58] showed that in assemblages of birds and fish containing species with less variable thermal niches (i.e. a low response diversity [59]), changes in abundance between years were more synchronized, and total community abundance less stable, compared with assemblages with higher thermal niche variability. If, as we suggest (figure 2*b*), species upper thermal limits are typically highly clustered within ecological assemblages, this implies that beyond a certain level of warming the number of species remaining within their thermal limits will decline steeply, and with it the potential for functional compensation.

## 3. The drivers of clustered warming tolerances

At the level of individual species, abrupt increases in exposure risk from increasing temperatures can arise because populations are clustered along thermal gradients in space (figure 2*a*). In theory this could simply be driven by physical geography, with most populations occurring under those conditions that are most common in the environment [60], what we term the ‘habitat availability hypothesis’. For example, Pigot *et al*. [35] showed that populations of a given species tend to cluster towards the hot edge of their realized thermal niche, reflecting the greater area of habitats at lower elevations (on land), and the relatively flat latitudinal temperature gradient within the tropics [61–63]. At a global scale, the uneven availability of thermal conditions, and thus patterns of occupancy within species’ thermal niches, arises inevitably from the spherical shape and rotation of the planet [61].

An alternative explanation for clustered warming tolerances across populations of a species, what we term the ‘habitat suitability hypothesis’, posits that species have higher rates of occupancy in those parts of environmental space where performance and rates of population growth are maximized. While the debate continues around whether abundance shows consistent patterns of variation across species’ ranges in different taxa [64], it is clear that abundance, and therefore suitability, is rarely uniform across space. For example, tropical reef fish abundances generally increase towards their Equator-ward range edge [21], possibly reflecting increased performance under hotter temperatures, until a threshold is reached [20]. In contrast, the distribution of abundances across most Alpine plant species ranges is cold-skewed, consistent with the idea that high elevation range limits are curtailed abruptly by a single limiting abiotic factor [65]. While both physical geography and physiology are likely to combine to shape the clustered occurrence of populations of species across environmental gradients, we are unaware of attempts to explicitly test the habitat suitability and habitat availability hypotheses together.

At the level of ecological assemblages, if nonlinear increases in risks of exposure to climate change can arise because of a clustering of species’ thermal limits (figure 2*b*), what then causes this clustering? In theory a clustering of thermal niche limits among co-occurring species could emerge if the species able to persist within local assemblages are strongly filtered by the thermal environment, and there is little thermal niche partitioning at local scales [66,67]. However, this cannot be the only driver because, across entire geographical regions, upper thermal limits often show markedly little variation between species (especially compared with lower thermal limits [68–71]) and are phylogenetically conserved [36,72,73]. It has been argued that this conservatism arises because, beyond some limited increases in niche breadth that can be achieved by local adaptation using existing or accessible alleles and genotypes, species’ thermal physiologies are constrained by hard limits to adaptation [73]. In this case, nonlinear increases in the risk of thermal exposure for ecological assemblages could arise from hard constraints to the mutational space accessible from genomes, or physical processes operating at the scale of molecules and cells [74].

The heat tolerance of a phenotype is the product of many physiological and biochemical mechanisms [75], any one of which could represent a hard limit for evolution. For example, temperature-induced lipid instability and resulting degradation of cellular membranes represent one possible constraint on upper thermal limits [69]. While lipid composition in some clades (such as Archaea) has evolved to tolerate extreme environments, the composition of the membranes of eukaryotes is less thermally flexible, which could present a hard limit to evolution [69]. Alternatively, the evolution of particular organ systems, rather than constraints on cellular biology *per se*, may create hard limits for species’ thermal limits. For example, the cardiovascular system has been demonstrated to be the limiting tissue in the survival of exothermic fish under extreme heat events [76].

In addition to hard limits to adaptation, the clustering of thermal limits may represent evolutionary convergence, where divergent taxa have adapted to conditions that are (or have been) more widespread and productive in space and over time [68,77,78], and have lost the capacity to tolerate conditions that are rarely, if ever, experienced [42]. In this case, by clustering thermal limits across species, the shape of the physical environment acts as an evolutionary attractor, causing nonlinear increases in the risk of thermal exposure for populations within a species across environmental gradients, as well as across populations of co-occurring species within assemblages. Understanding the relative importance of functional, evolutionary and adaptive constraints in causing clustered thermal niche limits and optima would help to quantify the capacity for species and their ecological assemblages to evolve a higher heat tolerance and so avoid nonlinear biodiversity losses under warming [72].

## 4. How biological responses can mediate the risks of nonlinear biodiversity losses

We suggest above that physiological, geographical and genomic constraints on phenotypes—operating across scales from proteins to planets—are expected to lead to a clustering of warming tolerances across species’ ranges, and within ecological assemblages. These clustered tolerances alone could drive a nonlinear increase in the exposure of populations to intolerable temperatures under climate warming, leading to accelerating declines in other facets of biodiversity, such as genetic [4], phylogenetic and functional diversity [79]. However, while local extinctions driven by warming will lead to a reduction in species’ geographical ranges, and in the species richness of ecological assemblages, these biodiversity losses could be offset by the dispersal of species to new locations. Indeed, range shifts and associated increases in species richness are, at least temporarily, being observed across many species and ecosystems [80–82].

In some instances, the dynamics of species losses arising from thermal exposure are likely to be the dominant factor in determining overall biodiversity change. For assemblages at the equatorial or low elevation end of thermal gradients, warmer-adapted species are unavailable to replace those that are lost [78,83,84]. Equally, for species at the cold edges of thermal gradients (e.g. on mountain tops), there is nowhere to move, so climate warming is expected to drive a net reduction in range size [85]. In contrast, for species and ecological assemblages at intermediate positions across thermal gradients, whether gains in range extent and abundance exceed losses will depend on rates of species dispersal, population establishment and growth [86], as well as on the plastic or adaptive shifts in the species on which they depend (see e.g. [54,87]).

Although rates of dispersal are likely to be insufficient to offset population losses for many species exposed to current rates of warming (e.g. long-lived and sedentary organisms, or those living in fragmented habitats [88–90]), an understanding of clustered warming tolerances could inform the capacity for dispersal among more mobile organisms (e.g. many pelagic marine species). For example, in the same way that the uneven availability of thermal conditions across space will drive a nonlinear contraction in a species’ geographical range, the same clustering would also cause nonlinear rates of range expansion as large areas with similar thermal conditions suddenly become suitable [90,91]. Equally, while the loss of resident species from ecological assemblages owing to climate warming is expected once their upper thermal limits are exceeded, the pool of potential new immigrants will expand as temperatures rise above the lower thermal limits of the species present in the surrounding region [92]. If species’ lower thermal limits are clustered, we would expect a nonlinear expansion of the potential species pool (or what could be termed an assemblage’s ‘immigration credit’ [93]), as the environment becomes suitable for many species near-simultaneously. However, the fact that upper thermal limits seem to be more phylogenetically conserved and less variable than lower thermal limits [68,71] suggests that the dynamics of immigration may be more linear and less abrupt than that of species’ loss from assemblages, at least in terms of the abiotic effects on species’ persistence. Projections of thermal opportunities and exposure across marine species are consistent with this prediction, showing that under future climate warming the risks of climate exposure accumulate more abruptly than do opportunities for range expansion from cooler regions [92].

**Figure 3.**
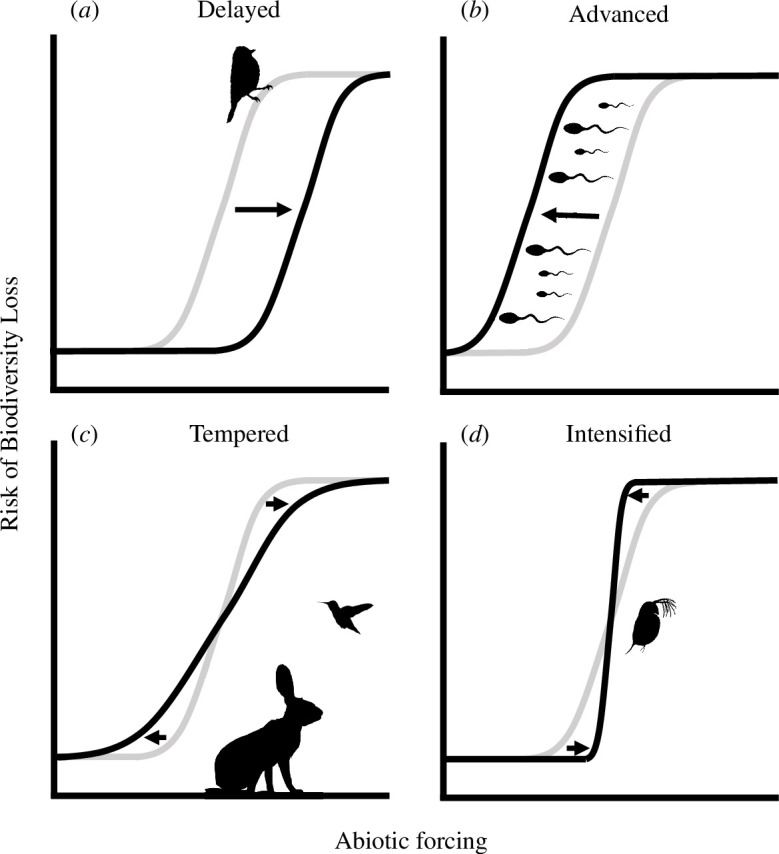
Ecological and evolutionary responses could mediate nonlinear increases in the risks of biodiversity loss. Grey lines denote the null expectation arising from clustered warming tolerances that are caused by geographical or physiological constraints. Black lines denote possible expectations for risk after including various ecological and evolutionary responses of populations to thermal exposure. Arrows indicate how risk changes. Abrupt responses can be (*a*) delayed (e.g. phenological plasticity delays the collapse of great tit (*Parus major*) populations owing to phenological plasticity [97]) or (*b*) advanced (e.g. in *Drosophila*, thermal limits for sperm are typically lower than for adults, causing risk to be underestimated if adult physiology is used to predict warming tolerances [98]). The abruptness of biodiversity responses can be (*c*) tempered (e.g. population thermal limits differ between sympatric mammal and bird species in the Mojave Desert owing to microhabitat use [99]) or (*d*) intensified (e.g. local adaptation of *Daphnia magna* to temperature variation across its latitudinal range causes reduced variation in warming tolerances [100]).

In addition to the potential for dispersal to buffer species and ecological assemblages (but see [88]), the units of biodiversity exposed to climate warming are also not fixed, immutable entities with no agency to change, or to affect their exposure. For example, genotypes are plastic in the phenotypes they create, and individuals can variously modify their behaviour, location and the timing of key life stages to avoid lethal temperatures (e.g. [74,75]). In addition, populations can evolve to adapt to novel conditions and changes in environmental variability [94,95]. Although the effects of these and other ecological and evolutionary processes on biodiversity are likely to be complex and often hard to predict [96], there are four ways that they could alter the null expectation of nonlinear dynamics of biodiversity loss driven by clustered warming tolerances. First, biological responses to environmental change could either (i) delay or (ii) advance the onset of biodiversity losses without altering the abrupt increase in how risk accumulates. Second, biological responses to environmental change could either (iii) temper or (iv) intensify the abruptness of biodiversity losses, making these more gradual or synchronized, respectively, but without altering the average timing of when these losses occur (figure 3). These categories are not mutually exclusive, and in the following sections we highlight how different ecological and evolutionary processes could alter both the onset and steepness of nonlinear biodiversity losses under rising temperatures.

### (a) Extinction lags in assemblages

When a population is exposed to conditions beyond its thermal limit, it should decline in abundance and eventually become locally extinct, in the absence of evolutionary rescue, or immigration from neighbouring populations. However, unless exposure is associated with the immediate and complete mortality of mature individuals, and a cessation of recruitment, this process will take time, resulting in an extinction lag [86,101]. Extinction lags are therefore expected to delay the onset of nonlinear contractions of species’ existing ranges and collapses of ecological assemblages. The pervasiveness of extinction lags has been used to argue against the notion that biodiversity will exhibit threshold responses to environmental change [102]. In fact, however, the effects of extinction lags in tempering or intensifying the nonlinearity of climate-driven biodiversity losses is unclear.

As the climate warms, a nonlinear increase in the number of populations exposed to unsuitable climates should also generate a nonlinear increase in extinction debt. However, whether this extinction debt leads to a delayed but nevertheless abrupt pulse of population losses depends on the variability in extinction lags across populations [93]. If populations and species experience different extinction lags (e.g. owing to demographic stochasticity or differences in ecology and life history [86,103]), this could spread out times to local extinction, tempering the abruptness of the collapse of species’ ranges and ecological assemblages. Alternatively, variation in species’ life histories, if correlated with their thermal limit, could intensify nonlinear responses, and make the payment of extinction debt abrupt. For example, species with ‘*k*-selected’ life history strategies can have longer extinction lags owing to their extended generation times [104], but also tend to have larger body sizes, a trait likely to be detrimental in warming environments owing to a smaller surface area to volume ratio [105]. In this case, large-bodied species whose thermal limits are reached earlier may take longer to decline, resulting in a stronger clustering in the timing of local extinctions than expected from the distribution of warming tolerances alone. These considerations mean that understanding how life history traits are linked to variation in thermal limits will be key for predicting how extinction lags alter the likelihood of abrupt climate-driven biodiversity losses [104].

### (b) Individual plasticity and evolutionary rescue within populations

While demographic processes can delay the timing of population extinction once thermal exposure occurs, plasticity, or the evolution of increased heat tolerance at a location, can enable a population to increase its upper thermal limit. In doing so, species could maintain a constant warming tolerance, avoiding declines and local extinction, provided the rates and magnitudes of environmental change do not exceed plastic limits or outpace adaptive change [94,95,106,107]. For example, great tits (*Parus major*) exhibit plasticity in breeding phenology, allowing them to track temporal shifts in the emergence of prey, potentially allowing them to reduce the abundance declines under future climate change that would be expected in the absence of plasticity or adaptation [97]. However, modelling suggests that, as warming continues, limits to plasticity will eventually be reached, and with rates of plasticity evolution insufficient to prevent increasing phenological asynchrony, the great tit population will collapse abruptly (figure 3*a*) [97]. Similarly, experimental transplants of *Senecio* daisies along an elevational gradient suggest that plasticity will prevent population decline, but only within the environmental conditions already experienced within their geographical range [108]. Such plasticity becomes much less effective when faced with novel environmental regimes, leading to rapid population declines, albeit alongside increases in genetic variance in plasticity and fitness that could lead to rapid evolutionary rescue [109].

In addition to delaying local extinction, whether plasticity and adaptive evolution generally temper or intensify the abruptness of species’ range losses and the collapse of ecological assemblages is unclear (figure 3). On the one hand, if populations that persist for longer can undergo more evolutionary change (perhaps also assisted by an increase in genetic variance in novel environments), this would act to broaden the distribution of warming tolerances and therefore reduce the abruptness of biodiversity loss in time and space. Similarly, if populations at the hot edge of a species’ range (i.e. with a narrow warming tolerance) have a lower capacity to increase their thermal limit (e.g. owing to hard physiological limits, behavioural ecology [110] or genetic variation [111]) than populations near the cold edge (i.e. with a larger warming tolerance), this would also cause population warming tolerances to become increasingly spread out over time, reducing the risk of abrupt range contractions. On the other hand, across species in a community absolute heat tolerance trades off against plasticity in heat tolerance; otherwise all species would be highly plastic to all environments [112]. While this trade-off should delay warming-driven population declines of the least heat-tolerant species as these are temporarily buffered by altering their phenotype, more heat-tolerant species do not experience the same benefits, thus compressing the variation in warming tolerance among species. In this case, plasticity ultimately delays the onset but intensifies the abruptness of biodiversity losses. Given these potentially opposing effects on the risk of abrupt biodiversity losses, understanding the strength and directions of correlations between the warming tolerance of populations and species and their plastic and adaptive capacity is thus a priority for future research.

### (c) Variation in population thermal limits across spatial gradients

**Figure 4.**
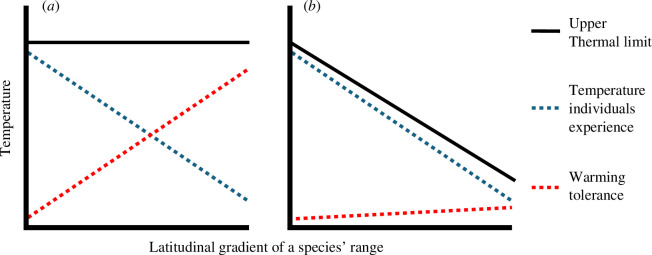
Without local adaptation in individual upper thermal limits (*a*) the population warming tolerances of a species will be greatest at the cold margin of their geographical range (e.g. at high latitudes). However, if populations are locally adapted to their thermal environment (*b*), warming tolerances will show little variation over the geographical range of a species. These clustered warming tolerances indicate that if the climate warms at the same rate everywhere, a locally adapted species will experience a more abrupt loss of populations over time, without invoking feedback loops or synchronous extreme weather events across the range.

Our discussion so far has assumed that all populations across a species’ geographical range share the same range-wide thermal limit. In this case, populations will differ in their warming tolerances across space, with these being the narrowest at the hot edge of the species’ range (figure 4*a*). However, this assumption could be unrealistic because evidence of local adaptation across space in species’ niche limits is widespread [113,114]. For example, to counteract lower oxygen concentrations at higher temperatures, tropical populations of the zooplankton *Daphnia magna* have evolved elevated haemoglobin levels compared with their temperate counterparts [100]. With strong local adaptation in space, populations will have thermal limits matching the conditions experienced by the individuals in each location [115]. As a result, while local adaptation across space drives variation in population thermal limits, it tends to equalize warming tolerances across populations [116] (figure 4*b*). The implication is that as the climate warms, in the absence of adaptive evolution, populations in colder locations will be exposed to intolerable conditions sooner than expected if all populations share the same range-wide upper thermal limit, causing a more abrupt wave of thermal exposure across the species’ range (figure 3*d*). Such adaptation to local climate regimes may explain why climate-driven population declines are already occurring across the entire geographical range of some species rather than only at the hot edges of their distributions [117,118].

While consistently strong local adaptation across space will tend to equalize population warming tolerances, spatial variation in the degree to which populations are locally adapted could have the opposite effect. Specifically, if adjacent populations share similar thermal environments but vary in their thermal limits (e.g. owing to one population experiencing gene flow from a cooler region), this will lead to greater differences in population warming tolerances than expected when assuming a single range-wide thermal limit, thus tempering risks of abrupt warming-driven biodiversity losses. In addition, if species exhibiting extensive spatial local adaptation can also adapt more rapidly to changing conditions over time, this could delay the onset of abrupt biodiversity losses in locally adapted species [119].

In addition to local adaptation, variation in environmental tolerance across space can arise owing to developmental plasticity [120], and for long-lived stationary organisms (e.g. trees) this could have a similar effect of intensifying risk to species’ ranges. For example, *Pinus edulis* trees in wetter parts of their range are more drought-sensitive than trees in drier locations, potentially owing to their development under less extreme conditions [121]. As a result, hydric tolerances are clustered among populations, all of which are at risk from increasing drought. In theory, plasticity could also enable individuals in what were historically wetter locations but now exposed to drier conditions, to develop more xerophytic phenotypes, expanding their hydric tolerances and thus tempering the risk of abrupt losses of populations of a species. For short-lived species, such developmental plasticity is generally expected to buffer populations from the negative effects of warming [97], assuming environmental cues remain reliable as climate regimes shift [122]. However, for long-lived organisms this buffering depends on the extent to which plasticity is bidirectional, and for many organismal traits (e.g. tree root architecture responses to drought [123]), a developmental plastic change in one direction may not be easily reversed. This lack of reversibility of some plastic traits may create a critical limit to the population resilience of long-lived species in the increasingly variable environments of the future [124].

As well as phenotypic plasticity, individuals can also be buffered against climatic change by altering their behaviour or local site occupancy to exploit climatic variation at fine spatial scales [125–129]. For example, North American mammals tend to use cooler habitats at the warm edge of their geographical ranges and warmer habitats at the cool edge of their ranges [125]. Similarly, European butterflies [126] and understorey plants [128] occupy warm microrefugia at the northern extents of their ranges. Such plasticity indicates an ability of individuals to exploit microclimate variation over short spatial scales, expanding the warming tolerance of the population when measured using coarse macroclimate data. This effect, operating across local populations, can act to delay predictions of population extinctions [130], until limits to microhabitat occupancy or availability are reached [131].

Although exploiting microclimate refugia can delay when a population is extirpated, paradoxically it could also advance the onset of when local populations experience adverse and sudden impacts from climate change. For example, species adapted to stable forest understorey conditions [132] have evolved to have reduced upper thermal limits [36] and narrower thermal niches [133,134], and thus can be highly vulnerable even though extreme temperatures are buffered below plant canopies [135,136]. Furthermore, if there is substantial fine-scale heterogeneity in climate over space then even in populations that (according to macroclimate data) appear to have a high warming tolerance, the hottest microsites could already be intolerably hot [137]. As the climate warms, these intolerably hot conditions will spread, and if individuals are constrained from accessing cooler microclimates, for example, owing to the availability of suitable soil types for plants or vegetation for animals, then mortality events or population declines should occur as the area of suitable microclimates shrinks. This means that even if we are concerned with the loss of individuals and decline of populations, rather than just local extinctions (i.e. when the last individual dies), accounting for microclimate variability could advance the predicted onset of abrupt increases in mortality risk across species’ geographical ranges [118].

As in the case of spatial variation in the capacity for adaptive evolution and plasticity discussed above, if the delay in local extinction due to microclimatic variation differs across a species’ geographical range (e.g. owing to differences in the extent of microclimate variability), this could variously temper or intensify how abruptly risk expands within and across species. Understanding how microclimate distributions will change in the future is, therefore, likely to be critical for predicting abrupt climate-driven biodiversity losses [138]. Furthermore, species that are able to avoid extreme body temperatures by behaviourally thermoregulating or exploiting fine-scale microclimatic variation should reduce (or at least delay) the selective pressure they experience for increased thermal limits [73,110]. Stasis in thermal limit evolution could therefore cause clustering in physiological thermal limits, but not necessarily warming tolerances, highlighting the need to incorporate behaviour into mechanistic models of future biodiversity dynamics [138,139].

### (d) Variation across species in thermal experience

Just as variation in thermal limits across populations within a species could either temper or intensify abrupt range contractions under climate warming, variation in the thermal conditions experienced by populations of different species at a single location could either temper or intensify the risk of abrupt species losses within ecological assemblages. For example, Riddell *et al*. [99] found a ubiquitous decline of bird species across a desert community in response to recent drought and warming, while mammals had generally persisted. These contrasting dynamics were attributed to the use of different microclimates by each taxon (e.g. the use of burrows by mammals) and their different energy demands. While this example suggests that differences in the thermal conditions experienced by species locally can temper abrupt responses across the community, so potentially maintaining ecological function (figure 3*c*), within each taxon there was little variation in response, with bird populations declining regardless of whether a species was specialized for desert conditions. This suggests that rather than reducing the intensity of abrupt biodiversity losses, variation in the thermal conditions experienced by species locally could alternatively lead to more abrupt losses than expected based on the distribution of species’ critical thermal limits.

Species are generally and by necessity well adapted to their environment. Dark-coloured species of ants dominate the hotter canopies of tropical rainforests [140], different species of temperate terrestrial isopods have higher desiccation resistance towards hot forest edges [141] and marine fishes living deeper in the water column are smaller [142]. As a result, the variation in critical thermal limits observed within ecological assemblages (e.g. figure 2*b*) could overestimate the variation in population warming tolerances across species. In other words, if species with higher upper thermal limits are exposed to hotter conditions this will tend to equalize species warming tolerances, and thus intensify the abruptness of biodiversity exposure under warming. Pincebourde & Casas [143] provide an example of this phenomenon in phyllophagous insects. These species have varied physiological limits, but using millimetre-resolution leaf temperature models, the authors found that these thermal limits positively covary with the temperatures they experience. As a result, although species have a diversity of thermal limits, and exploit different microhabitats, warming tolerances are tightly clustered so that risks of thermal exposure for all species in the assemblage are expected to increase synchronously as leaf temperatures rise.

Just as individuals can move in space to exploit cooler microclimates (or thermally tolerant species are forced into space where better competitors cannot follow), individuals can also move in time, by altering their daily activity patterns or their phenology. The capacity for, or rate of, phenological shifts can vary across populations and species [144]. For example, butterflies have a variety of life history traits, such as the number of generations they produce each year [145], that can mediate the ability of species to shift their phenology in response to climate change. Such variation could temper the risk of abrupt collapse in ecological assemblages because, even if species exhibit similar thermal limits, some species will be relatively buffered from change as they track suitable temperatures through time, essentially spreading out population warming tolerances across species. However, these differences in phenology may already reflect, and be driven by, interspecific differences in thermal limits (e.g. less heat-tolerant species emerge earlier in spring). In this case, despite the appearance of varied thermal limits, all species could actually have similar warming tolerances and be equally at risk from climate change.

Although in the above we have assumed that organisms are thermal conformers, passively mirroring the environmental temperatures that surround them, key aspects of organismal biology have evolved to alter individual exposure to the thermal environment. For example, morphological phenotypes alter the amount of solar radiation that individuals absorb as thermal energy [146], while behavioural thermoregulation or the generation of metabolic heat can allow species to increase or decrease their body temperatures in accordance with their thermal optima [110]. Such decoupling between environmental temperatures and body temperatures could spread out warming tolerances, and therefore temper the abruptness of biodiversity declines. However, the opposite could also be true: it could cause many phenotypes to be experiencing the same thermal environment, independently of regional or local climatic variation. For instance, in a similar way to how fine-scale temperature variations experienced by phyllophagous insects are tightly matched to their physiology [143], ectotherm body temperatures are likely to positively covary with thermal limits. Indeed, tropical butterflies with lighter colours express lower heat tolerance [147], presumably owing to their decreased absorption of infrared radiation and, thus, relatively low body temperatures [146]. Such active alteration of the individual’s thermal experience by phenotype means that warming tolerances estimated incorporating morphology and behaviour could be more clustered than expected if we consider physiological limits in isolation.

## 5. Clustered warming tolerances across scales, from proteins to the planet

So far, we have focused on species’ geographical ranges and ecological assemblages. However, clustered warming tolerances could also drive abrupt increases in risk across a much broader range of levels of biological organization. At the scale of a single cell, abrupt declines in cellular function due to clustered warming tolerances of molecules occur if the different proteins within the cell share similar denaturation temperatures, as is expected from a biophysical perspective [148]. For an organ or individual, a threshold leading to organ failure or death, respectively, can arise where cells and organs experience similar conditions and exhibit a clustering of their thermal limits [37] (figure 2*c*). Similarly, populations may be at risk of abrupt collapse where a small additional change in conditions exceeds the environmental tolerance of a disproportionate number of individuals. At each level of biological organization, from cells to ecological assemblages, nonlinear increase in risk to the system arises through the clustering of warming tolerances in the level beneath.

Moving beyond local assemblages of co-occurring species, an expectation that different biomes will exhibit different sensitivities to environmental change could suggest that there is unlikely to be a single sharp threshold in the risk to global biodiversity as a function of the magnitude of global warming [16,18]. For example, Trisos *et al.* [12] showed that even though clustering of thermal limits of co-occurring species will cause abrupt increases in the proportion of species at risk of thermal exposure within 100 km assemblages, at a global scale there is a relatively smooth, albeit nonlinear, increase in the number of species at risk, because these collapses occur at different levels of warming in different assemblages. However, variation in thresholds across regions and ecosystems does not rule out nonlinear increases in risk, because some areas and systems support a disproportionate number of species, and some species play critical roles in the functioning of multiple ecosystems (e.g. seasonally migrating birds [149]). In addition, variation in rates of warming in space could mean that divergent thermal limits are reached at similar levels of global warming. For instance, tropical species are thought to be closest to their thermal limits [46], with recent evidence suggesting terrestrial temperate species are underfilling the warmer end of their niches [150]. While this would be expected to temper the abruptness of biodiversity loss, faster warming at high latitudes (‘polar amplification’ [151]) or high elevations could cause a greater clustering in when thermal limits are exceeded across latitudes. Indeed, climate-related mortality events and local extinctions are already well documented in temperate regions, suggesting that some species in those systems are also approaching the limits of their adaptive capacity [97,152].

## 6. Anticipating clustered warming tolerances to mitigate biodiversity loss

Predicting where populations, species and ecosystems are at risk of collapse due to the short-term pulses or the long pressure of climate change, and when ecosystems will shift abruptly into new (and less productive) states, is critical for targeting our efforts to bend the curve of biodiversity loss. As a universal driver of biological systems, we have focused on the nonlinear risks to biodiversity that arise from changes in temperature, rather than other aspects of the abiotic environment, and independently of the varied ways in which individuals and species interact. Some of these aspects will be strongly correlated with temperature, such as drought risk on land and oxygen availability in aquatic ecosystems, whereas others will vary independently (e.g. nutrient availability or habitat fragmentation). Accurate predictions of biodiversity responses therefore require considering species’ limits and risks of exposure across multiple niche dimensions. Nevertheless, we expect that the general patterns we have identified for temperature will also hold for other niche dimensions, given abrupt responses are expected wherever populations exhibit clustering in tolerances to any abiotic change.

Understanding how prevalent clustered warming tolerances are will require more comprehensive data on how abiotic limits are distributed both across populations within species and across species that co-occur in assemblages. Although some studies exist (e.g. [46,48,73]), data on physiological tolerances across multiple populations of a given species, or for complete assemblages, are still relatively sparse across both taxa and space. Studies that do exist often use different methodologies to collect data, which can alter estimates of thermal limits and so reduce their comparability [153]. Although standardized methods could alleviate this issue [153], there are fundamental difficulties in comparing physiological limits at broader taxonomic scales [154]. For example, invertebrate thermal limits are often measured by ascertaining knockdown behaviour [36], whereas tree eco-physiologists assay photosynthetic activity rates [45], two distinct limits that may represent different parts of the thermal performance curve. There is also growing evidence for sublethal effects of elevated temperature on fitness, such as impacts on fertility [98], and a recognition that chronic (rather than acute) thermal exposure also drives population dynamics in wild populations [155]. Similarly, individuals can exhibit changing temperature performance relationships across their lifetimes [156], particularly when inhabiting diverse thermal environments during distinct life stages, as observed in most insect species [157,158]. This means that, although the critical thermal limits required to knockdown or kill adult individuals have been found to predict the response of species to local climates [36,72], the thermal limits of populations may be significantly lower (figure 3*b*).

In addition to the more comprehensive sampling of abiotic limits, reliably determining warming tolerances and the strength of clustering depends on accurately estimating the contemporary and future conditions encountered by organisms. Ignoring the variation in conditions experienced by organisms over fine spatial and temporal scales could risk either overestimating [99] or underestimating [143] threshold responses for biodiversity. Fortunately, better niche limit estimates, advances in microclimatic sensing and modelling, and biophysical modelling of body temperatures [139], are all rapidly increasing researchers’ abilities to predict the fitness effects of climate change on organisms [154].

We have discussed nonlinear risks to biodiversity arising as a result of clustered warming tolerances and dynamic feedbacks (e.g. tipping points driven by species interactions) as distinct phenomena, but both could simultaneously drive abrupt responses to environmental change across different levels of biological organization. For example, Doughty *et al*. [37] identified a possible tipping point in tropical forests driven by critical leaf temperature limits. According to this model, leaf death in rainforest canopies arising from heatwaves results in a decrease in evaporative cooling, further driving up the temperature experienced across individual trees, eventually resulting in tree death [37]. However, the results of Doughty *et al*. also show that leaf temperatures are strongly clustered (figure 2*c*). This would imply that, as temperatures increase, the amount of time that leaves are exposed to critical temperatures will increase nonlinearly, representing a clustered distribution of warming tolerances for individual leaves, in addition to the tipping point dynamics observed. Moving from an individual tree to the entire planet, recent syntheses suggest that different Earth system tipping elements (e.g., Amazon Forest dieback) will be triggered at similar global warming levels (figure 2*d*) [38]. While many of these tipping elements are physical (rather than biological) and slow-onset (e.g. West Antarctic ice sheet collapse), this suggests that abrupt changes in the biosphere are likely to arise as a combination of both tipping point dynamics and the clustered warming tolerances of these tipping elements. A key question for biosphere-level dynamics is whether phylogenetically distinct but functionally equivalent systems (e.g. Asian versus African versus Amazonian rainforests) have similar warming tolerances.

We suspect that many nonlinear biodiversity responses to abiotic change are caused by both tipping points (driven by feedbacks in a system) and clustered warming tolerances (driven by the distribution of biological units across thermal gradients, or thermal limits within niche space). Distinguishing between these and partitioning their relative effects is a priority for future research owing to their contrasting predicted consequences for the dynamics of biodiversity loss and recovery. Systems characterized by tipping points can fail to recover even once the abiotic forcing is removed owing to self-reinforcing feedback loops. By contrast, risks to biodiversity from clustered warming tolerances should stop increasing once forcing ceases, albeit with the caveat that there may be delays before the full extent of loss is apparent (owing to lagged responses), and that local biodiversity losses may be irreversible over timescales meaningful to human society.

Tipping points driven by feedbacks are hard to predict in systems characterized by more than a handful of species with well described interactions [25]. The prediction of regime shifts often requires long-term monitoring data, but even with such data, there may be few signs of change before irreversible transitions begin [159]. In contrast, it may be simpler to predict species or assemblages at risk of abrupt collapse due to clustered warming tolerances because in theory this requires only data on the distribution of niche limits, or on the abiotic conditions populations are exposed to through space and time. Although not trivial to ascertain, niche limits have long been estimated from macroecological (e.g. [36]) or eco-physiological data (e.g. [103]), while the development of physical models [160] alongside cheap, small and battery-efficient sensors [161] is enabling the prediction and characterization of climate at fine scales in space and time [162,163]. Combining these data with climate projections could lead to improved forecasts of where, when and how abruptly species and ecological assemblages will cross critical abiotic limits under climate change.

## 7. Conclusion

The recent and rapid erosion of Earth’s biodiversity poses an existential threat to human wellbeing. We have argued that the risk to biodiversity is expected to increase at an accelerating rate with rising temperatures owing to the non-uniform distribution of biodiversity across environmental gradients which manifests in a clustering of population warming tolerances within and across species. This clustering arises from physiological, geographical and genomic constraints to evolutionary divergence and looks likely to drive sudden and widespread losses of existing biodiversity, even in the absence of the kinds of feedback loops that are usually invoked to explain the abrupt collapse of populations, species and ecosystems. A critical question is to understand how biological responses to changing climates mediate these nonlinear and potentially abrupt shifts. We suggest that while some ecological and evolutionary processes may delay and temper the risks of abrupt collapse of species and ecosystems, others could advance their onset and increase their intensity. Biological processes, such as plastic shifts or evolutionary rescue, that are currently buffering ecological systems against climate change may be like the steep front of the beach, only temporarily slowing the advancing tide of climate change (albeit with some early inundation driven by the rough seas of thermal variation around the mean). Ultimately, the buffering effect of the steep foreshore will only delay, and potentially intensify, the inevitable biodiversity collapse. An improved understanding of the drivers, ubiquity and effects of clustered warming tolerances would identify those geographical regions, species and ecosystems that are most at risk from abrupt biodiversity loss, and where and when these losses will occur under different warming scenarios. Understanding and predicting these nonlinear warming risks is essential to inform species’ conservation and ecosystem management, and where efforts should be focused on maintaining species and community resilience in space and time. Enhancing those features of biodiversity (e.g. functional, phylogenetic and genomic diversity) and the physical environment (e.g. microclimatic variation and buffering) that expand and spread out population warming tolerances should buy time for biological processes (e.g. dispersal and evolutionary adaptation) and management interventions to mitigate the risks of an abrupt attrition of biodiversity, and the irreversible loss of a productive and socially just future.

## Data Availability

All data used in this article (for figure 2) are available through the original papers that are cited in the figure caption.
